# An anthropometric model to estimate neonatal fat mass using air displacement plethysmography

**DOI:** 10.1186/1743-7075-9-21

**Published:** 2012-03-21

**Authors:** Andrea L Deierlein, John Thornton, Holly Hull, Charles Paley, Dympna Gallagher

**Affiliations:** 1 New York Obesity Nutrition Research Center, St. Luke's-Roosevelt Hospital, New York, NY, USA; 2 Department of Preventive Medicine, Mt. Sinai School of Medicine, New York, NY, USA; 3 Department of Dietetics & Nutrition, University of Kansas Medical Center, Kansas City, KS, USA; 4 Department of Pediatrics, St. Luke's-Roosevelt Hospital, New York, NY, USA; 5 Institute of Human Nutrition, Columbia University, New York, NY, USA

**Keywords:** Neonate, Fat mass, Anthropometry, Air displacement plethysmography

## Abstract

**Background:**

Current validated neonatal body composition methods are limited/impractical for use outside of a clinical setting because they are labor intensive, time consuming, and require expensive equipment. The purpose of this study was to develop an anthropometric model to estimate neonatal fat mass (kg) using an air displacement plethysmography (PEA POD^® ^Infant Body Composition System) as the criterion.

**Methods:**

A total of 128 healthy term infants, 60 females and 68 males, from a multiethnic cohort were included in the analyses. Gender, race/ethnicity, gestational age, age (in days), anthropometric measurements of weight, length, abdominal circumference, skin-fold thicknesses (triceps, biceps, sub scapular, and thigh), and body composition by PEA POD^® ^were collected within 1-3 days of birth. Backward stepwise linear regression was used to determine the model that best predicted neonatal fat mass.

**Results:**

The statistical model that best predicted neonatal fat mass (kg) was: -0.012 -0.064*gender + 0.024*day of measurement post-delivery -0.150*weight (kg) + 0.055*weight (kg)^2 ^+ 0.046*ethnicity + 0.020*sum of three skin-fold thicknesses (triceps, sub scapular, and thigh); R^2 ^= 0.81, MSE = 0.08 kg.

**Conclusions:**

Our anthropometric model explained 81% of the variance in neonatal fat mass. Future studies with a greater variety of neonatal anthropometric measurements may provide equations that explain more of the variance.

## Background

The ability to assess neonatal body composition is essential for understanding how fetal exposures to nutrients, hormones, and environmental factors relate to infant nutritional status, growth, and the development of diseases later in life. Current validated neonatal body composition methods are impractical for use outside of a clinical setting because they are labor intensive, time consuming, and require expensive equipment. Few anthropometric models to estimate neonatal body composition within the first few days post-delivery have been developed. Dauncey and colleagues [1] and Westrate and Deurenberg [2] were among the first to develop anthropometric models that could be applied to neonates and young infants; however, these models were based on theoretical body composition equations and were not validated in neonates. More recently, Catalano and colleagues [3] and Schmelzle and Fusch [4] developed anthropometric models to estimate neonatal fat mass using total body electric conductivity (TOBEC) and dual energy x-ray absorptiometry (DXA), respectively. Though these models were validated they were based on previous technology that had not been validated specifically for use in neonates and infants.

The PEA POD^® ^Infant Body Composition System is an infant-sized air displacement plethysmography system that directly measures infant body weight and volume and uses these values to derive body fat percentage, fat mass, and fat-free mass [5]. This system has been validated in infants against the gold standard four compartment model [6] and deuterium dilution [7]. The purpose of the current study was to develop an anthropometric model to estimate neonatal body fat mass based on body composition assessments from the PEA POD^® ^among a healthy, term, multi-ethnic infant population. This model may be a useful research tool for evaluating body fat in similar newborn populations when more sophisticated body composition measurement methods are unavailable.

## Methods

### Participants

Women were recruited while inpatient on the maternity floors of St. Luke's-Roosevelt Hospital located in New York, New York. Those with term (gestational age > = 37 weeks), singleton infants with no known birth defects, congenital abnormalities, or admissions to the NICU were included. Infants of women diagnosed with gestational diabetes mellitus, hypertension, or pre-eclampsia were not included. Data from 128 infants were available for analysis. The study was approved by the Institutional Review Board at St. Luke's-Roosevelt Hospital. A written consent was obtained from a parent prior to participation in the study.

Information on infant race/ethnicity was determined by maternal self-report using the following four categories: non-Hispanic White, non-Hispanic Black, Hispanic, and Asian. Women were asked to select a race/ethnic category for each of the infant's parents and grandparents. When all selected categories were within the same race/ethnicity, the infant was classified as that race/ethnicity. When multiple race/ethnicity categories were selected, the infant was classified as "Other".

### Anthropometric measurements

All infant measurements were conducted prior to hospital discharge between days 1 and 3 post-delivery by highly trained study personnel. Measurements were not collected on day 0 ( < 24 hours post-delivery) because pilot data from our laboratory suggest that there is an initial weight loss in infants during this time period [8]. Among infants included in the pilot study (n = 8), the mean (SD) weights measured during days 0, 1, and 2 post-delivery were 3286 (680) g, 3163 (669) g, and 3136 (682) g, respectively. Based on repeated measures analysis of variance, mean weight measured at days 1 and 2 were significantly less than the mean weight measured at day 0 (*p *< 0.0001) while there was no difference between mean weights measured at days 1 and 2 (*p *= 0.27) [8].

Infant length at birth was measured to the nearest 0.1 cm using an infant length board (Shorr Productions). Abdominal circumference (below the umbilicus) was measured using disposable measuring tapes. Harpenden calipers (British Indicators, Sussex, England) were used to measure skin-fold thicknesses in the biceps, triceps, sub scapular, and thigh regions. Skin-fold thickness measurements were made by lifting the skin with the thumb and index finger. All skin-fold thickness measurements were taken twice. If there was a difference greater than 2 mm between the two measurements, a third measurement was taken. The two measurements in agreement were averaged and used for analyses.

### Body composition assessment

The PEA POD^® ^Infant Body Composition System Version 3.1.0 (Life Measurement Instruments, Concord, CA) was used to measure infant body weight and volume. The system was calibrated each day prior to infant testing; a calibration cylinder with known volume was used to calibrate the chamber and a 2 kg weight was used to calibrate the scale. Testing procedures have been previously described in detail [5]. Briefly, the infant was undressed and a standard tight fitting hat (Allentown Scientific Associates, INC) was placed on its head to minimize the amount of air trapped in its hair (necessary for body volume assessments). Any items on the infant that could not be removed, such as the umbilical clamp or identification bracelets, were used to tare the scale for body weight and volume measurements. The infant was then placed on the scale and the body weight was measured to the nearest 0.10 g. Following the body weight measurement, the infant was placed inside the chamber of the PEA POD^® ^and a body volume measurement was obtained, lasting approximately 2 minutes. Body volume and weight measurements were then used to calculate body density, which was used to derive fat and fat-free masses based on the sex-specific equations developed by Foman and colleagues [9]. Studies have shown that the PEA POD^® ^is a valid tool to measure percent body fat when compared to the gold standard 4 compartment (4 C) model and deuterium dilution [6,7]. There were no differences found between the percent body fat measurements by the PEA POD^® ^and the 4 C model or the deuterium dilution [6,7]. The mean age of the infants in these prior validation studies was approximately 8 weeks and ranged from 0.4-23.0 weeks, which is older than the current study population.

### Statistical analysis

Stata 11.0 was used for all statistical analyses. Backward stepwise linear regression was used to determine the statistical model that best predicted neonatal fat mass. The full model included all candidate variables: infant gender, race/ethnicity, gestational age (weeks), day of measurement post-delivery (days), weight (kg), length (cm), abdominal circumference (mm), and skin-fold thickness (mm) measurements. Dummy variables were created for the categorical variables, gender and race/ethnicity, and used as independent variables in the regression equations. Nonlinear relationships were also considered using products, logarithms, and powers of the independent variables and logarithm of the dependent variable. Residual analyses were performed to assess the fit of the model. Scatter plots of the residuals versus the predicted values and scatter plots of the residuals versus the independent variables were examined to determine if there were any patterns that would indicate the model required modification. Also, the hypothesis that the distribution of the residuals was consistent with the normal distribution was tested using the Shapiro-Wilk normal test statistic.

## Results and discussion

Distributions of the baseline characteristics for the infants stratified by gender (male, n = 68 and female, n = 60) are shown in Table 1. The mean gestational age of the sample was approximately 39.5 weeks. All anthropometric measurements were similar between male and female infants; however, male infants had greater average birth weight and length and lower average percent body fat compared to female infants. Sub scapular skin-fold thickness was most strongly correlated with fat mass followed by weight, triceps skin-fold thickness, and thigh skin-fold thickness (Table 2). Length and biceps skin-fold thickness were modestly to weakly correlated with fat mass. Using stepwise regression analysis neonatal fat mass (kg) was predicted using the following variables: gender, Hispanic ethnicity (Hispanic vs. non-Hispanic), day of measurement post-delivery, weight, and sum of three skin-folds, triceps, sub scapular, and thigh (Table 3). The coefficient of determination, R^2^, was 0.81 (*p *< 0.0001) meaning that 81% of the variability in fat mass was accounted for by the statistical model. The standard error, root mean square error, was 0.08 kg.

**Table 1:** Distributions of infant characteristics stratified by infant gender (n = 128)

Characteristic		Boys (n = 68)		Girls (n = 60)
	n	Frequency (%)	n	Frequency (%)
Race/Ethnicity				
Caucasian	30	44.1	23	38.3
African American	2	2.9	6	10.0
Hispanic	13	19.1	12	20.0
Asian	7	10.3	6	10.0
Other	16	23.5	13	21.7
Age at Measurement (days)				
1	27	39.7	29	48.3
2	32	47.1	25	41.7
3	9	13.2	6	10.0
		Mean (SD)	n	Mean (SD)
Gestational Age (weeks)	68	39.2 (1.2)	60	39.0 (1.2)
Weight (kg)	68	3.4 (0.5)	60	3.2 (0.4)
Length (cm)	65	52.3 (2.4)	54	50.1 (3.3)
Triceps Skinfold Thickness (mm)	68	4.4 (1.1)	60	4.5 (1.1)
Biceps Skinfold Thickness (mm)	68	3.3 (0.8)	60	3.1 (0.7)
Sub scapular Skinfold Thickness (mm)	68	3.9 (1.1)	60	3.8 (0.9)
Thigh Skinfold Thickness (mm)	68	6.4 (1.8)	60	6.4 (1.9)
Fat Mass (kg)	68	0.4 (0.2)	60	0.4 (0.2)
Percent Body Fat (%)	68	12.1 (4.1)	60	13.2 (3.8)

**Table 2:** Correlations of infant anthropometric and fatness variables (n = 128)

	Fat Mass	Percent Fat	Weight	Length	Triceps SFT*	Biceps SFT	Sub scapular SFT	Thigh SFT
Fat Mass	1.00							
Percent Fat	0.95	1.00						
Weight	0.72	0.53	1.00					
Length	0.48	0.30	0.70	1.00				
Triceps SFT	0.70	0.66	0.53	0.33	1.00			
Biceps SFT	0.32	0.23	0.38	0.20	0.35	1.00		
Sub scapular	0.73	0.66	0.56	0.38	0.70	0.37	1.00	
SFT								
Thigh SFT	0.64	0.61	0.48	0.35	0.68	0.40	0.62	1.00

*SFT, Skin-fold Thickness

**Table 3:** Regression coefficients with standard errors and P values for independent variables* in final model with infant fat mass (kg) as dependent variable

**Independent Variable**	**Coefficient**	**Standard Error**	***P***
SConstant	-0.012	0.246	0.962
Infant Gender	-0.064	0.014	< 0.001
Ethnicity	0.046	0.017	0.010
Age at Measurement (days)	0.024	0.010	0.023
Weight (kg)	-0.150	0.146	0.306
Weight^2 ^(kg^2^)	0.055	0.022	0.013
Sum of Skin-fold Thicknesses (mm)	0.020	0.003	< 0.001

R^2 ^= 0.81, MSE = 0.08 kg

* Infant gender: 1 = male and 0 = female; Ethnicity: 1 = Hispanic and 0 = not Hispanic; Age at measurement: number of days post-delivery; Weight: weight at measurement (kg); Weight^2^: weight at measurement squared (kg^2^); Sum of Skin-fold Thicknesses: sum of triceps, sub scapular, and thigh skin-fold thicknesses (mm)

In this study we evaluated the ability of anthropometric measurements to predict neonatal body fat as measured by the PEA POD^®^, an infant-sized air displacement plethysmography system, in a multiethnic population of term infants. The results showed that our anthropometric model, which included infant gender, race/ethnicity, age, weight, and skin-fold thickness measurements, explained 81% of the variance in neonatal fat mass.

Skin-fold thickness and other anthropometric measurements have long been incorporated into models to predict body fat mass and percent fat; however, there are few models that have been developed for use in neonates. Dauncey and colleagues [1] used a combination of sub scapular and triceps skin-fold thicknesses, circumferences, and limb length measurements to estimate body volume and derive percent fat. Westrate and Deurenberg [2] related the sum of the biceps, triceps, sub scapular, and suprailiac skin-fold thicknesses to total body density and estimated total body fat percentage using age and gender-specific equations on the relation between body fat percentage and body fat density. The use of these anthropometric models is limited because they were based on theoretical body composition models and not validated using methods specific for neonates. In one study, the Dauncey model was found to only moderately correlate with neonatal fat mass measured by TOBEC (R^2 ^= 0.54, *p *= 0.0001) [3]. Catalano and colleagues (1995) developed an anthropometric model based on TOBEC to estimate neonatal fat mass in a multiethnic sample of 194 infants. The model that was most highly correlated with fat mass included birth weight, flank (suprailiac) skin-fold thickness, and length (R^2 ^= 0.78, *p *= 0.0001) and was prospectively validated in a sample of 65 infants (R^2 ^= 0.84, *p *= 0.0001) [3]. Similarly, Schmelzle and colleagues correlated skin-fold thicknesses with fat mass measurements from DXA in neonates and young infants [4]. In a sample of 104 white, predominantly term infants measured at 0, 2 and 4 months of age, they found that an exponential equation including the sum of four skin-fold thicknesses (triceps, biceps, suprailiac, and sub scapular) and length best predicted fat mass (R^2 ^= 0.948, *p *< 0.001). The model was validated using a bootstrap sampling method (R^2 ^= 0.936), however, infant body weight was not considered as a possible predictor of fat mass [4].

In the current study, we correlated measurements of fat mass from the PEA POD^® ^with anthropometric measurements in neonates taken within 1-3 days post-delivery. We found that infant weight, triceps, sub scapular, and thigh skin-fold thicknesses as well as infant age at measurement (in days), Hispanic ethnicity, and gender were significant predictors of fat mass. Our model was able to predict fat mass with a coefficient of determination (R^2^) of 0.81. This value is similar but slightly lower than the R^2 ^values reported in previous studies [3,4] and may in part be explained by differences in methodologies used across the studies. For example, available skin-fold thickness measurements varied between the studies and we lacked a measure of suprailiac skin-fold thickness. Suprailiac as well as sub scapular skin-fold thickness represents a measure of central adiposity while biceps, triceps, and thigh skin-fold thicknesses represent peripheral adiposity [10]. It is possible that the addition of suprailiac skin-fold thickness to our model would improve its predictability. Additionally, the reference method for assessing neonatal body composition differed across studies. TOBEC is a previous body composition method that has been replaced with more recent technologies such as DXA and air displacement technology, which is used in the PEA POD. Both TOBEC and DXA are not specific for use in infants (although, very recently pediatric software has become available for use with iDXA) and their accuracy within this population is of some concern [11] compared to the PEA POD. The PEA POD has been validated for use in infant populations slightly older than the one used in the current study [5-7]. Slight differences in fat mass estimates between these three technologies may have influenced the predictability of the anthropometric models.

Our model is based on a population of term infants from healthy pregnancies and is not representative of small (i.e. preterm, low birth weight, or growth-restricted) and large (i.e. macrosomic) infants. Though our sample included some small and large infants and there was a wide distribution of percent fat, ranging from approximately 3-24%, the majority of infants had a percent fat between 7% and 17% (10^th ^percentile = 7.3% and 90^th ^percentile = 17.6%). It is unclear how well anthropometric models are able to predict percent fat or fat mass in small and large infants. Skin-fold thickness measurements provide information regarding the amount of subcutaneous fat in both central (truncal) and peripheral (limbs) regions; however, they do not provide any information on visceral (intra-abdominal) fat. Small and large infants may have varying amounts of visceral versus subcutaneous fat relative to normal weight infants [3], which may limit the ability of our anthropometric model to predict fat mass in these infants.

## Conclusions

We found that an anthropometric mcbodel including infant gender, age, race/ethnicity, weight, and skin-fold thickness measurements explain 81% of the variability in fat mass measured by the PEA POD in a multi-ethnic population of term infants at 1-3 days post-delivery. Currently, there are a limited number of anthropometric models to estimate body fat during early infancy. These models may be useful for identifying at-risk infants or for evaluating percent fat or fat mass on a group level. Future studies that include comprehensive and more complete anthropometric measurements, a reference body composition method specific for use in infants (such as the PEA POD), and a large multi-ethnic sample with adequate numbers of small and large infants are necessary.

## Competing interests

Each author declared that she or he has no conflict of financial or personal interests in any company or organization sponsoring this study.

## Authors' contributions

ALD was involved in data analysis and manuscript writing. JT was involved in data analysis and manuscript writing. HH conceived the manuscript concept, was involved in study coordination, data collection, and manuscript editing. CP was involved in data collection. DG was involved in study design, manuscript writing, provided administrative support, and supervision. All authors read and approved the final manuscript.
